# Alginate oligosaccharide alleviates D‐galactose‐induced cardiac ageing via regulating myocardial mitochondria function and integrity in mice

**DOI:** 10.1111/jcmm.16746

**Published:** 2021-07-06

**Authors:** Wenjing Feng, Jianya Liu, Shan Wang, Yi Hu, Hui Pan, Ting Hu, Huashi Guan, Dongfeng Zhang, Yongjun Mao

**Affiliations:** ^1^ Department of Geriatric Medicine The Affiliated Hospital of Qingdao University Qingdao China; ^2^ Department of Epidemiology and Health Statistics The School of Public Health of Qingdao University Qingdao China; ^3^ Key Laboratory of Marine Drugs Ministry of Education Shandong Provincial Key Laboratory of Glycoscience and Glycotechnology School of Medicine and Pharmacy Ocean University of China Qingdao China; ^4^ Marine Biomedical Research Institute of Qingdao Qingdao China; ^5^ Laboratory for Marine Drugs and Bioproducts of Qingdao National Laboratory for Marine Science and Technology Qingdao China

**Keywords:** alginate oligosaccharide, cardiac ageing, D‐galactose, mitochondria, oxidative stress

## Abstract

Ageing is a crucial risk factor for the development of age‐related cardiovascular diseases. Therefore, the molecular mechanisms of ageing and novel anti‐ageing interventions need to be deeply studied. Alginate oligosaccharide (AOS) possesses high pharmacological activities and beneficial effects. Our study was undertaken to investigate whether AOS could be used as an anti‐ageing drug to alleviate cardiac ageing. D‐galactose (D‐gal)‐induced C57BL/6J ageing mice were established by subcutaneous injection of D‐gal (200 mg·kg^‐1^·d^‐1^) for 8 weeks. AOS (50, 100 and 150 mg·kg^‐1^·d^‐1^) were administrated intragastrically for the last 4 weeks. As a result, AOS prevented cardiac dysfunction in D‐gal‐induced ageing mice, including partially preserved ejection fraction (EF%) and fractional shortening (FS%). AOS inhibited D‐gal‐induced up‐regulation of natriuretic peptides A (ANP), brain natriuretic peptide (BNP) and ageing markers p53 and p21 in a dose‐dependent manner. To further explore the potential mechanisms contributing to the anti‐ageing protective effect of AOS, the age‐related mitochondrial compromise was analysed. Our data indicated that AOS alleviated D‐gal‐induced cardiac ageing by improving mitochondrial biogenesis, maintaining the mitochondrial integrity and enhancing the efficient removal of impaired mitochondria. AOS also decreased the ROS production and oxidative stress status, which, in turn, further inhibiting cardiac mitochondria from being destroyed. Together, these results demonstrate that AOS may be an effective therapeutic agent to alleviate cardiac ageing.

## 1 INTRODUCTION

Ageing can be broadly defined as the time‐dependent accumulation of cellular damage, which causes the progressive functional decline of tissues and organs.^1^ In general, age‐related changes in heart mainly include left ventricular hypertrophy, increased myocardial fibrosis and cardiac insufficiency. Ageing is a crucial risk factor for the development of age‐related cardiovascular diseases, which leads to a markedly increased prevalence of cardiovascular diseases in the ageing population.^2^, ^3^ Thus, more effective treatment strategies are urgently needed for the treatment of cardiac insufficiency caused by ageing.

Alginate is a natural seaweed‐derived polysaccharide extracted from brown algae. Alginate oligosaccharide (AOS) is produced by depolymerizing alginate, which has multiple unique advantages such as water‐soluble, non‐toxic, non‐immunogenic and biodegradable.^4^ AOS exerts promising biological activities. This oligosaccharide possesses neuroprotective,^5^ hypolipidaemic,^6^ anti‐oxidative,^7^ anti‐inflammatory,^8^ anti‐aggregatory,^9^ anti‐tumour,^10^ anti‐bacterial^11^ and immunoregulatory properties,^12^ as well as suppress obesity^13^ and advanced glycation end‐product (AGE) activities.^4^ Notably, no studies have been explored the ability of AOS to protect against cardiac ageing.

Mitochondria are double‐membraned organelles and serve multiple functions in cellular bioenergetics and metabolism.^14^ The heart is a high‐energy demand and high mitochondrial content organ. Cardiac mitochondria generate 90% of the ATP and play a vital role in maintaining a healthy heart. Mitochondrial dysfunction, which can accelerate ageing process in mammals, is one of the most important mechanisms of ageing.^15^, ^16^ Age‐related mitochondrial dysfunction shows different features including loss of mitochondrial integrity, reduced efficiency of mitochondrial biogenesis, defective quality control by autophagy, decreased mitochondrial membrane potential (MMP), alterations in mitochondrial dynamics, increased reactive oxygen species (ROS) formation and accumulation of mutations in mitochondrial DNA (mtDNA).^17^ Due to the lower biogenesis and reduced clearance, the combination of increased damage and reduced renewal in mitochondrial may eventually contribute to the ageing process.^18^, ^19^ In addition, the progressive mitochondrial dysfunction results in increased production of ROS and oxidative stress, which in turn cause further mitochondrial deterioration and cellular damage and ultimately contribute to age‐related cardiac dysfunction.^20^

D‐galactose (D‐gal)‐induced ageing animal models have been widely used to study ageing mechanisms and anti‐ageing effects of drugs.^21^ Chronic administration of a large amount of D‐gal causes cell metabolism disorders and cell injury which accelerate the ageing process and eventually cause structural and functional changes in cardiovascular system.^22^ Therefore, in the current study, we established the D‐gal‐induced mouse ageing model to systemically evaluate the anti‐cardiac ageing activity of AOS and further investigate the possible underlying mechanism.

## 2 MATERIALS AND METHODS

### 2.1 Drugs and reagents

D‐galactose was obtained from Sigma‐Aldrich. AOS was purchased from Qingdao BZ Oligo Biotech Co., Ltd. Malondialdehyde (MDA) assay kit was obtained from Jiancheng Bioengineering Institute. Rabbit polyclonal antibodies against BNP, PGC‐1α, p67‐phox and gp91‐phox were obtained from Abcam Inc. Rabbit monoclonal antibodies against natriuretic peptides A (ANP) and SIRT3 and mouse monoclonal antibody against p53 were obtained from Abcam Inc. Rabbit polyclonal antibodies against beclin‐1 and p‐mTOR were purchased from Cell Signaling Technology. Rabbit polyclonal antibody against p21 and mouse monoclonal antibody against p47‐phox were obtained from Santa Cruz Biotechnology, Inc. Rabbit polyclonal antibodies against mTOR were purchased from Proteintech Group, Inc. Mitochondrial extraction kit and JC‐1 mitochondrial membrane potential assay kit were purchased from Beijing Solarbio Science & Technology Co., Ltd. TIANamp Genomic DNA kit was obtained from Tiangen Biotech (Beijing) CO., LTD. Mouse mtDNA probe PCR kit was purchased from Beijing Tiandz Gene Technology CO., Ltd. Goat Anti‐Rabbit IgG (H + L) (peroxidase/HRP conjugated) and Goat Anti‐Mouse IgG (H + L) (peroxidase/HRP conjugated) were purchased from Elabscience Biotechnology Co., Ltd. All other chemicals and reagents were purchased from standard commercial suppliers unless otherwise specified.

### 2.2 Animals

Male 8‐week‐old C57BL/6J mice (20 ± 2 g bodyweight, n = 40) were obtained from Jinan Pengyue Experimental Animal Breeding Co., LTD. All mice were fed with normal diet and housed in a 12‐hour light/12‐hour dark cycle under specific pathogen–free (SPF) conditions at the Laboratory Animal Center of the Medical Department of Qingdao University. All animal experimental procedures complied with the ‘Guide for the Care and Use of Laboratory Animals’ published by the US National Institutes of Health and Public Health Service policy on Humane Care and Use of Laboratory Animals. The study protocols were approved by the Committee on the Ethics of Animal Experiments of Qingdao University (approval number: QYFYWZLL25840).

### 2.3 D‐gal‐induced ageing model and drug administration

The mice were randomly divided into five groups (n = 8 each group): control, D‐gal, D‐gal + low‐dose AOS (D‐gal + AOS‐50), D‐gal + middle‐dose AOS (D‐gal + AOS‐100) and D‐gal + high‐dose AOS (D‐gal + AOS‐150) groups. Ageing was induced in C57BL/6J mice through subcutaneous injection of D‐gal (200 mg/kg, Sigma‐Aldrich) for 8 weeks.^23^, ^24^ The control mice were given subcutaneous injection of equivalent sterile water. Four weeks after the injection of D‐gal, the D‐gal + AOS‐50, D‐gal + AOS‐100 and D‐gal + AOS‐150 groups were intragastrically administered with AOS (50, 100 and 150 mg·kg^‐1^·d^‐1^) for another 4 weeks, respectively. The control group and D‐gal group mice received intragastrical administration of equivalent saline.

### 2.4 Echocardiography

Echocardiography was performed on anaesthetized mice by using a Vevo2100 (VisualSonics) with a 30‐MHz transducer at the end of the experiment. The following parameters were measured as previously described: left ventricular ejection fraction (EF) and left ventricular fractional shortening (FS).^25^

### 2.5 Histological analysis

The heart samples were fixed with 4% paraformaldehyde for 24 hours and then embedded in paraffin and cut into 4‐μm‐thick sections. H&E and Masson's trichrome staining were used to visualize the cardiac architecture and cardiac fibrosis, respectively. The slices were examined under an inverted light microscope (TE 2000, Nikon). Corresponding data were analysed and calculated by investigators who were blinded to treatment group assignment.

### 2.6 Transmission electron microscopy

The ultrastructure of mitochondria was determined using transmission electron microscopy analysis. Following the previous study,^26^ fresh left ventricular tissues were cut into 1 mm^3^ blocks and fixed in 2.5% glutaraldehyde for 2 hours at 4℃. After post‐fixed in 1% osmium tetroxide for 2 hours at room temperature, left ventricular tissues were dehydrated then embedded in resin. Serial ultrathin sections (30‐40 nm) were collected onto copper grids and finally stained with 0.5% uranyl acetate followed by 1% lead citrate. The ultrastructural analysis of stained sections was determined under a Tecnai G2 12 transmission electron microscopy (FEI Co.).

### 2.7 Mitochondrial membrane potential (MMP) Determination

Mitochondrial membrane potential was measured using the JC‐1 immunofluorescent staining and flow cytometry. Fresh heart tissues were used to prepare frozen sections or extract mitochondria. Frozen sections were used for JC‐1 immunofluorescent staining according to the manufacture's protocol. Fluorescence was determined at excitation/emission 485/580 nm (red) and excitation/emission 485/530 nm (green) using a fluorescence microscope.

In addition, mitochondria were isolated from fresh heart tissue using the mitochondrial extraction kit according to the method published by Peng et al^27^ Extracted mitochondria were then used to detect the integrity of the MMP through measuring the fluorescent intensity of JC‐1. Briefly, mitochondria were incubated with JC‐1 in JC‐1 Assay Buffer for 10min at room temperature. Carbonyl cyanide m‐chlorophenylhydrazone was applied to disrupt MMP and served as positive control following the manufacturer's instructions. The samples were analysed at 488 and 575 nm by flow cytometry using the CellQuest Software (Becton Dickinson).

### 2.8 Measurement of mtDNA copy number

Mitochondrial DNA copy number was measured by quantitative real‐time PCR (qPCR). In short, heart tissues were homogenized and removed proteins and RNAs using proteinase K and RNaseA. The total DNAs were extracted by TIANamp Genomic DNA kit and then quantified by QuickDrop Spectrophotometer from Molecular Devices (Holliston, MA). Mouse mtDNA probe PCR kit was used to amplify the sample's mtDNA by Fluorescence PCR Detection System (Hangzhou Bioer Technology Co. Ltd). mtDNA copy number was calculated from the qPCR positive control curve according to the manufacturer's protocol.

### 2.9 In situ detection of ROS production

As described previously, DHE fluorescence staining was performed to evaluate the in situ level of ROS production.^28^ Briefly, fresh heart tissues were embedded into OCT compound and then immediately frozen using a cryostat at −20℃. The frozen heart tissues were cut into 6‐μm‐thick histological slides. Heart slices were washed with 1×phosphate‐buffered saline (PBS) and then incubated with DHE (5 μmol/L) solution in a light‐protected humidified chamber at 37℃ for 30 minutes. After incubation, transverse heart sections were rinsed with 1×PBS. Finally, images were obtained with a fluorescence microscope, and fluorescence intensity was quantified by ImageJ software by a blind tester.

### 2.10 Lipid peroxidation measurement

As the major secondary oxidation products of lipid peroxidation, the concentration of malondialdehyde (MDA) in serum and heart homogenates was measured according to the instruction of MDA Assay kit (Jiancheng Bioengineering Institute).

### 2.11 Western blot analysis

Heart tissues were homogenized in RIPA buffer containing phosphatase and protease inhibitors and centrifuged at 12 000 rpm at 4℃ for 20 minutes. Then, the supernatant was collected for subsequent experiments. Protein concentration was determined using a BCA protein assay kit. Equal amounts of proteins were placed in 10% SDS‐PAGE gel and transferred to PVDF membranes (Roche). The membranes were blocked with the sealing solution for 1 hour and incubated with corresponding primary antibodies at 4℃ overnight. Then, membranes were washed in TBS‐T and incubated with the appropriate secondary antibodies. Subsequently, membranes were washed in TBS‐T and visualized using the ECL Western blotting detection reagents (Bio‐Rad). The protein expression was quantified by densitometry analysis of bands using ImageJ software. The housekeeping protein GAPDH was used as loading control. The dilution ratios of the primary and secondary antibodies were shown as follows: ANP (1:1000), BNP (1:2000), p53 (1:1000), p21 (1:1000), PGC‐1α (1:1000), SIRT3 (1:1000), beclin‐1 (1:1000), p‐mTOR (1:1000), mTOR (1:1000), p47‐phox (1:500), p67‐phox (1:1000), gp91‐phox (1:2000), GAPDH (1:2000), Goat Anti‐Rabbit IgG (H + L) (peroxidase/HRP conjugated) (1:5000) and Goat Anti‐Mouse IgG (H + L) (peroxidase/HRP conjugated) (1:5000).

### 2.12 Statistical analysis

Data were expressed as mean ±SEM. Statistically significant was determined with the one‐way ANOVA followed by Student‐Newman Keuls (SNK)’s post hoc test. *P* < .05 was considered to be statistically significant.

## 3 RESULTS

### 3.1 Effects of AOS on cardiac function and the expression of ageing markers in D‐gal‐induced ageing mice

**FIGURE 1:**
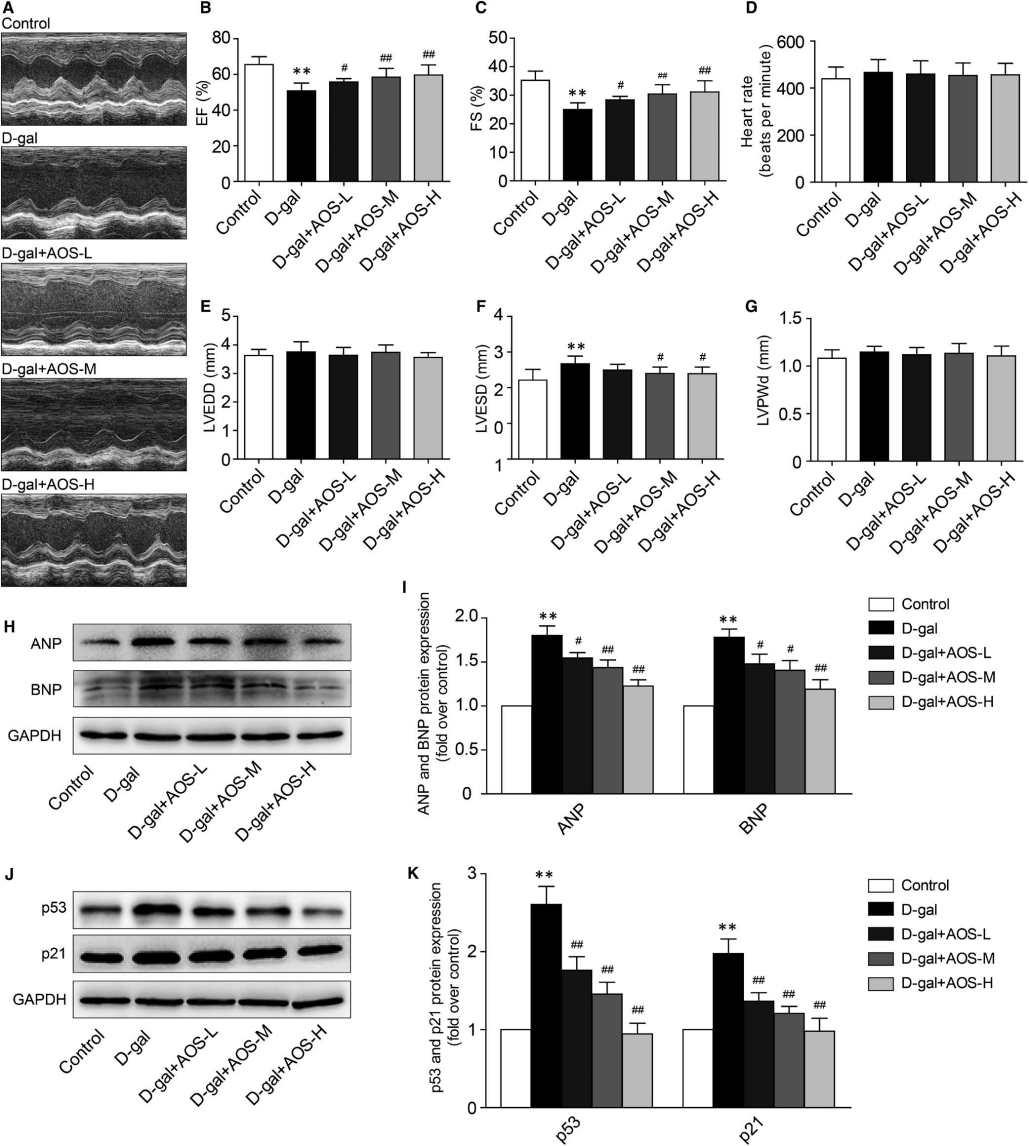
Alginate oligosaccharide prevented D‐gal‐induced cardiac alterations in C57BL/6J mice. (A) Representative echocardiographic M‐mode images from mice with D‐gal and AOS treatment. (B‐G) Ejection fraction (EF)%, fraction shortening (FS)%, heart rate, left ventricular end‐diastolic diameter (LVEDD), left ventricular end‐systolic diameter (LVESD) and left ventricular posterior wall thickness at end‐diastole (LVPWd) were measured by echocardiography. (H) The expression of ANP and BNP in heart tissues was detected by Western blot. (I) The corresponding quantitative data of (D). (J) The expression of ageing markers p53 and p21 in heart tissues was determined by Western blot. (K) The corresponding quantitative data of (D). AOS‐L, AOS‐M and AOS‐H represent 50, 100 and 150 mg·kg^‐1^·d^‐1^ of AOS, respectively. **P < .01 vs Control mice, #*P* < .05 vs D‐gal mice, ##*P* < .01 vs D‐gal mice

Echocardiography was used to analyse the cardiac function of mice. D‐gal‐induced ageing mice showed a significant decrease in EF% and FS%. However, AOS significantly prevented cardiac dysfunction in D‐gal‐induced ageing mice, including partially preserved EF% and FS% (Figure 1A‐C). As shown in Figure 1D‐G, there was no statistical difference in heart rate between each group. D‐galactose mice had a tendency to increase LVEDD (left ventricular end‐diastolic diameter) and LVPWd (left ventricular posterior wall thickness at end‐diastole), but there was no statistically significant difference compared with the control mice. However, LVESD (left ventricular end‐systolic diameter) was significantly increased in D‐gal‐induced ageing mice, and AOS decreased LVESD in a dose‐dependent manner. The expression of ANP and BNP in heart tissue was determined by Western blot to further verify the cardiac function of mice in each group. As shown in Figure 1H‐I, the levels of ANP and BNP were significantly increased in D‐gal‐induced ageing mice, and AOS significantly decreased the ANP and BNP levels in a dose‐dependent manner. These data indicated that AOS prevented cardiac dysfunction in D‐gal‐induced ageing mice. Furthermore, we detected the protein expression of ageing markers p53 and p21. As expected, p53 and p21 protein expression was significantly increased in heart tissues of D‐gal‐induced ageing mice, and AOS significantly decreased the p53 and p21 expression in a dose‐dependent manner (Figure 1J‐K).

### 3.2 Effect of AOS on cardiac histopathological changes in D‐gal‐induced ageing mice

**FIGURE 2:**
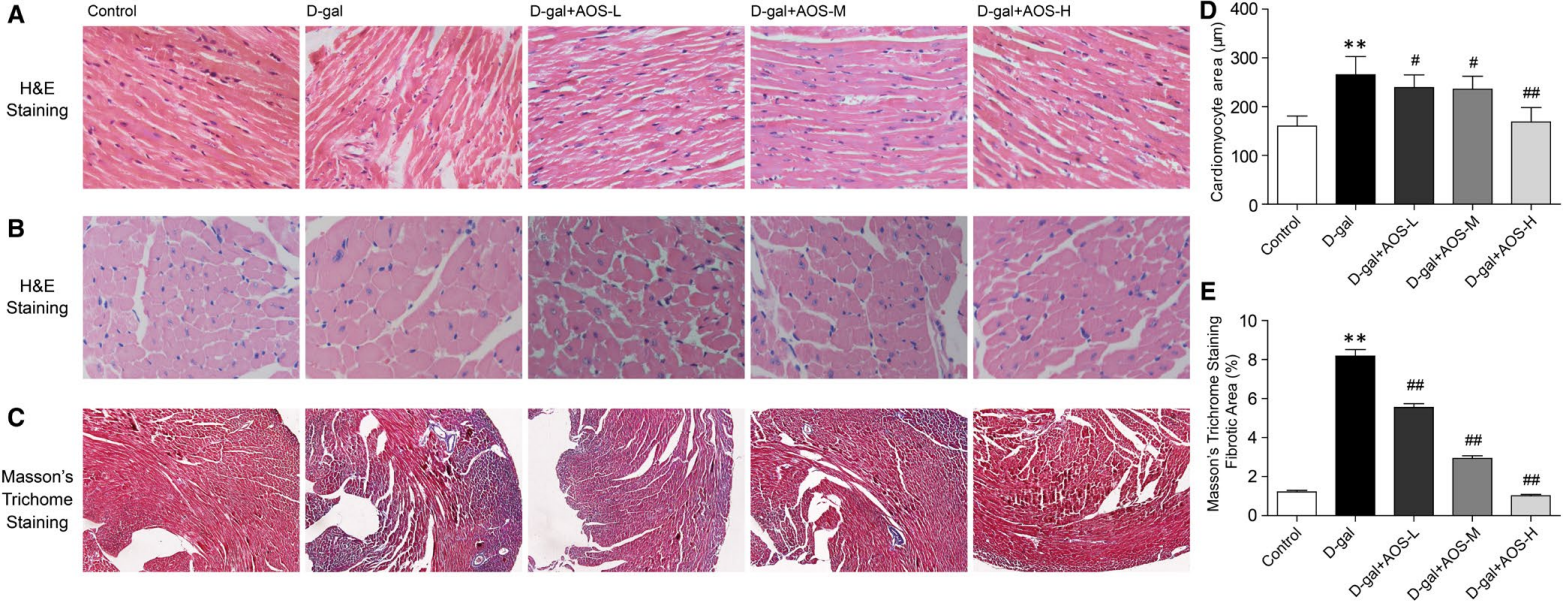
Alginate oligosaccharide treatment prevented the cardiac histopathological changes and cardiac fibrosis in D‐gal‐induced ageing mice. (A‐B) Morphological changes were assessed by H&E staining. (Magnification ×400) (C) The degree of cardiac fibrosis was investigated by Masson's trichrome staining. (Magnification ×100) (D‐E) The corresponding quantitative data of (B‐C). **P < .01 vs Control mice, #*P* < .05 vs D‐gal mice, ##*P* < .01 vs D‐gal mice

To investigate the myocardial architecture changes of mice, heart tissue slides were stained with haematoxylin and eosin (H&E). As shown in Figure 2A, compared with the control mice, D‐gal‐induced ageing mice exhibited abnormal myocardial architecture, mainly characterized by disordered arrangement of cardiomyocytes and increased intercellular space between cells. AOS administration significantly reduced the disordered arrangement and space between cardiomyocytes. H&E staining also demonstrated that left ventricular cardiomyocyte area was significantly increased in D‐gal‐induced ageing mice, and AOS treatment significantly decreased the cardiomyocyte area in a dose‐dependent manner (Figure 2B,D).

In order to clarify the degree of cardiac fibrosis, Masson's trichrome staining of heart cross sections was performed. As shown in Figure 2C,E, collagen accumulation in myocardium interstitial and perivascular regions was dramatically increased in D‐gal‐induced ageing mice. However, compared with the D‐gal group, AOS treatment significantly decreased the percentage of collagen area in a dose‐dependent manner (Figure 2C,E). These data suggested that AOS prevented D‐gal‐induced morphological changes and cardiac fibrosis in C57BL/6J mice.

### 3.3 Effect of AOS on the cardiac mitochondrial ultrastructure of D‐gal‐induced ageing mice

**FIGURE 3:**
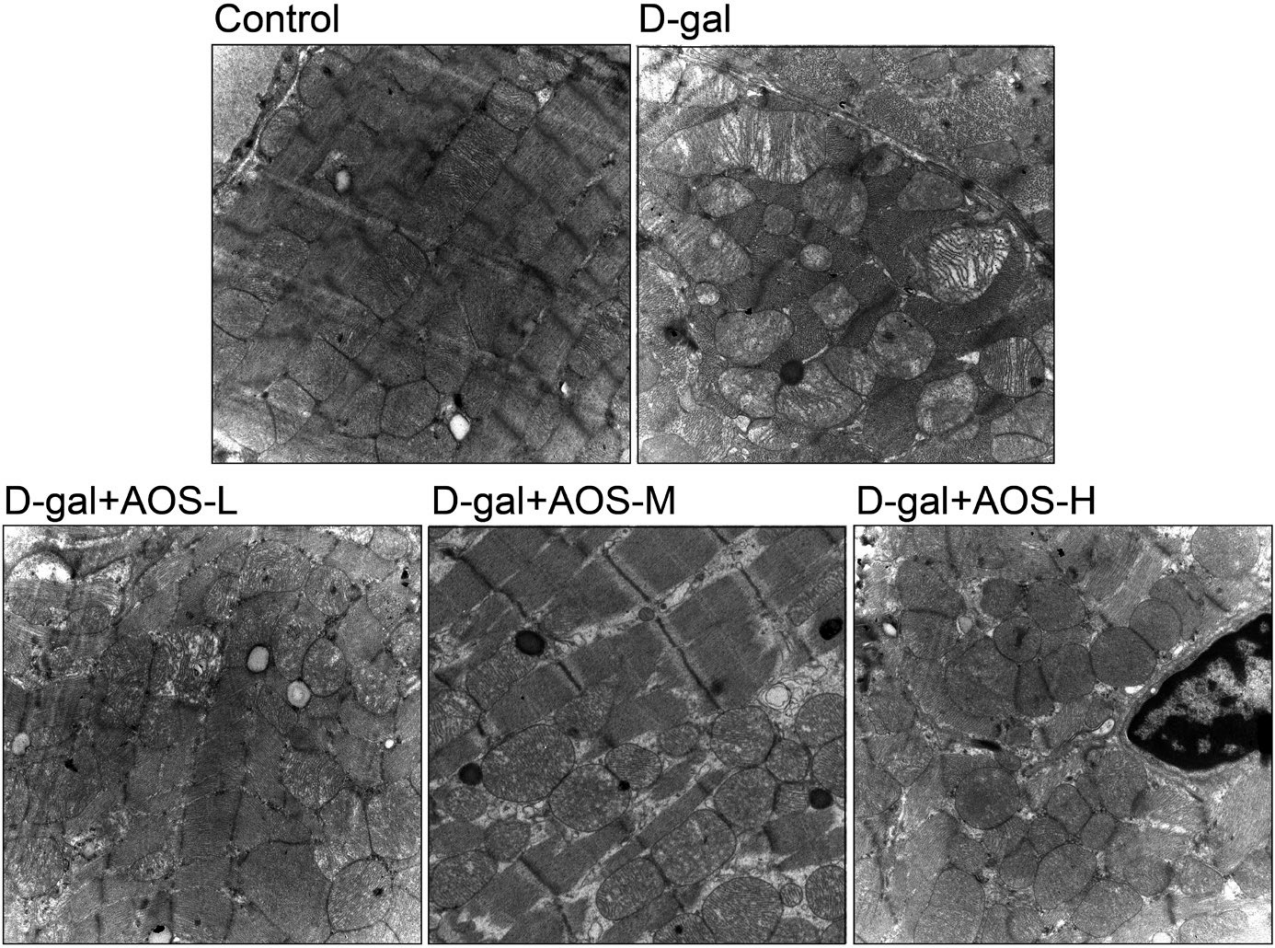
Alginate oligosaccharide treatment prevented the cardiac mitochondrial ultrastructure from being destroyed in D‐gal‐induced ageing mice. Cardiac mitochondrial ultrastructure was detected by transmission electron microscopy. (Magnification ×20 000)

We next used transmission electron microscopy to detect the alteration of cardiac mitochondrial ultrastructure. As shown in Figure 3, the cardiomyocyte mitochondria of control mice were structurally normal and showed clearly discernible cristae and electron‐lucent matrix. However, some cardiomyocyte mitochondria of D‐gal mice were enlarged, swelling and partial loss of cristae, and AOS treatment prevented the destruction of the cardiac mitochondrial ultrastructure in D‐gal‐induced ageing mice.

### 3.4 Effect of AOS on the cardiac MMP of D‐gal‐induced ageing mice

**FIGURE 4:**
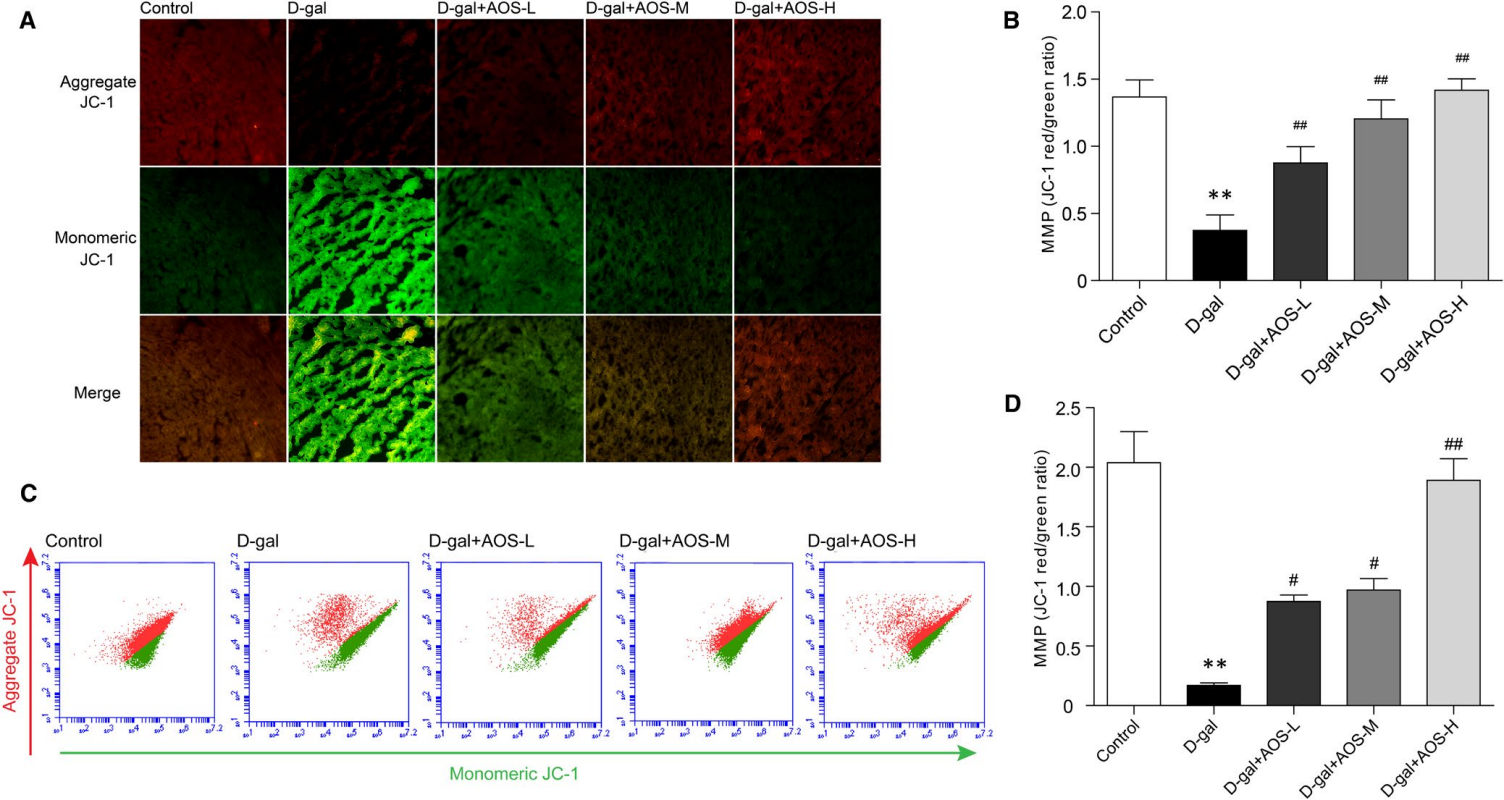
Alginate oligosaccharide treatment protected against D‐gal‐mediated decline of MMP. (A‐B) MMP was quantitatively determined by JC‐1 fluorescence staining. (C‐D) MMP was detected following quantification of flow cytometry analysis on JC‐1 staining. **P < .01 vs Control mice, #*P* < .05 vs D‐gal mice, ##*P* < .01 vs D‐gal mice

As an important determinant of functional state of mitochondria, MMP was measured using JC‐1 fluorescence staining and flow cytometry. In general, when mitochondria membrane potential is high, JC‐1 aggregates in mitochondria and emits red fluorescence, while JC‐1 exists as a monomer in cytosol and emits green fluorescence when mitochondria with low membrane potential.^29^ So the ratio of red fluorescence to green fluorescence could reflect the intensity of MMP. As shown in Figure 4A‐B, JC‐1 fluorescence staining result indicated that the level of cardiac MMP in D‐gal‐induced ageing mice was dramatically lower than that in control mice, and AOS administration protected against D‐galactose‐mediated decline of MMP. In addition, flow cytometry data indicated similar change trends in the MMP between groups after incubation with JC‐1 dye (Figure 4C‐D).

### 3.5 Effect of AOS on mitochondrial biogenesis and autophagy protein expression in the hearts of D‐gal‐induced ageing mice

**FIGURE 5:**
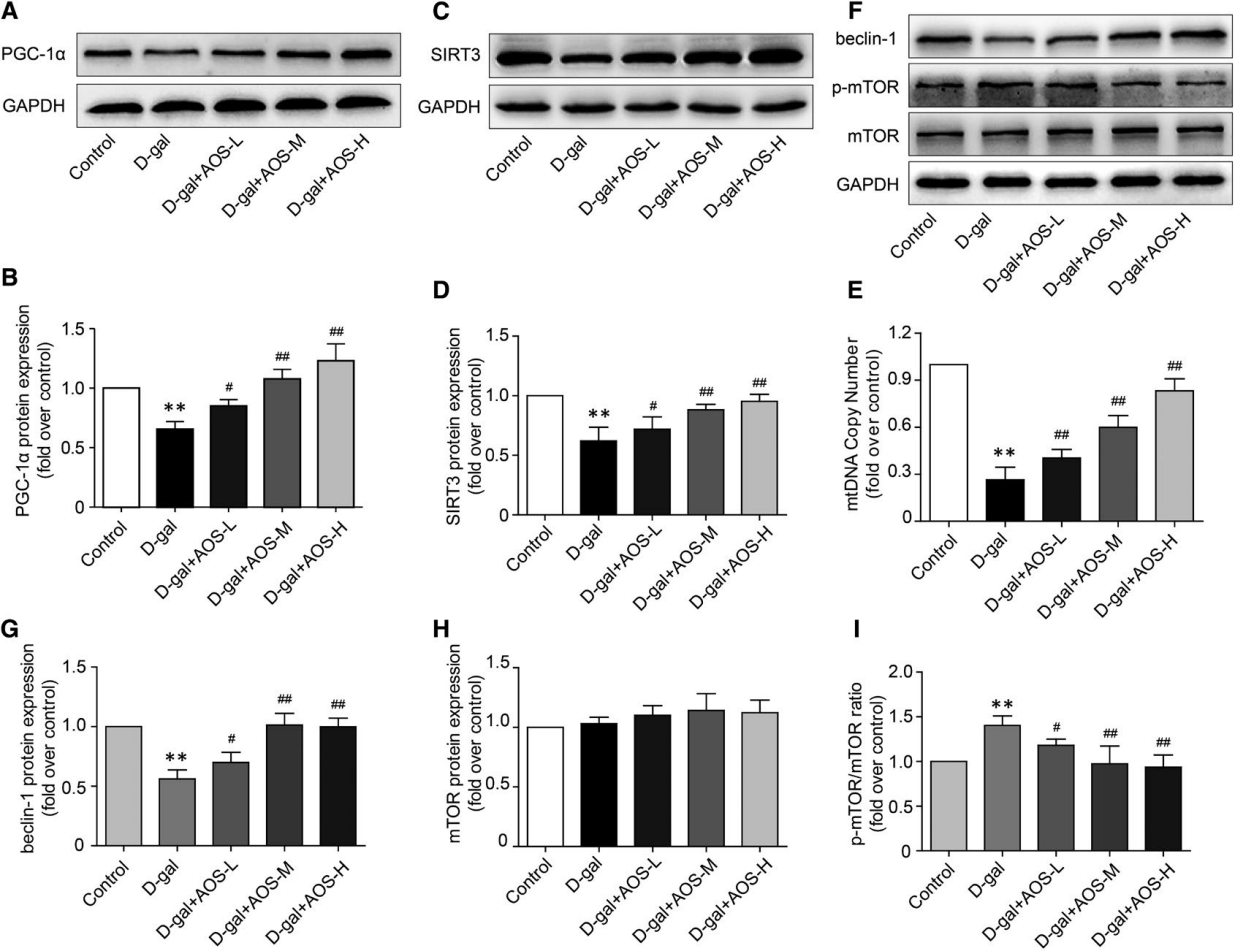
Alginate oligosaccharide up‐regulated the mitochondrial biogenesis and autophagy in D‐gal‐induced ageing mice. (A) The expression of mitochondrial biogenesis marker PGC‐1α in heart tissues was determined by Western blot. (B) The corresponding quantitative data of (A). (C) The expression of SIRT3 in heart tissues was determined by Western blot. (D) The corresponding quantitative data of (C). (E) MtDNA copy number was measured by mtDNA probe qPCR. (F) The expression of beclin‐1 and mTOR and phosphorylation of mTOR were detected by Western blot. (G‐I) The corresponding quantitative data of (F) **P < .01 vs Control mice, #*P* < .05 vs D‐gal mice, ##*P* < .01 vs D‐gal mice

We used Western blot to determine the protein expression of mitochondrial biogenesis marker PGC‐1α. As expected, a significant decrease in PGC‐1α protein expression was observed in heart tissues of D‐gal‐induced ageing mice, and AOS significantly increased the PGC‐1α expression in a dose‐dependent manner (Figure 5A‐B). Studies have shown that SIRT3 plays an important role in maintaining mitochondrial bioenergetics. Therefore, we further detected the SIRT3 expression in heart tissues. Our result showed that a significant decrease in SIRT3 protein expression in D‐gal‐induced ageing mice, and AOS significantly increased SIRT3 protein expression in a dose‐dependent manner (Figure 5C‐D). Moreover, mtDNA probe PCR result showed that mtDNA copy number was significantly decreased in D‐gal‐induced ageing mice, and AOS significantly increased the mtDNA copy number in a dose‐dependent manner (Figure 5E). These data indicated that AOS up‐regulated the mitochondrial biogenesis in D‐gal‐induced ageing mice.

We next used Western blot to determine the expression and phosphorylation of critical autophagy regulators in heart tissues. As shown in Figure 5F‐I, a significant decrease in beclin‐1 protein expression and a significant increase in mTOR phosphorylation were observed in heart tissues of D‐gal‐induced ageing mice. As expected, AOS significantly increased the beclin‐1 expression and decreased the mTOR phosphorylation in a dose‐dependent manner.

### 3.6 Effect of AOS on ROS production and oxidative stress in the hearts of D‐gal‐induced ageing mice

**FIGURE 6:**
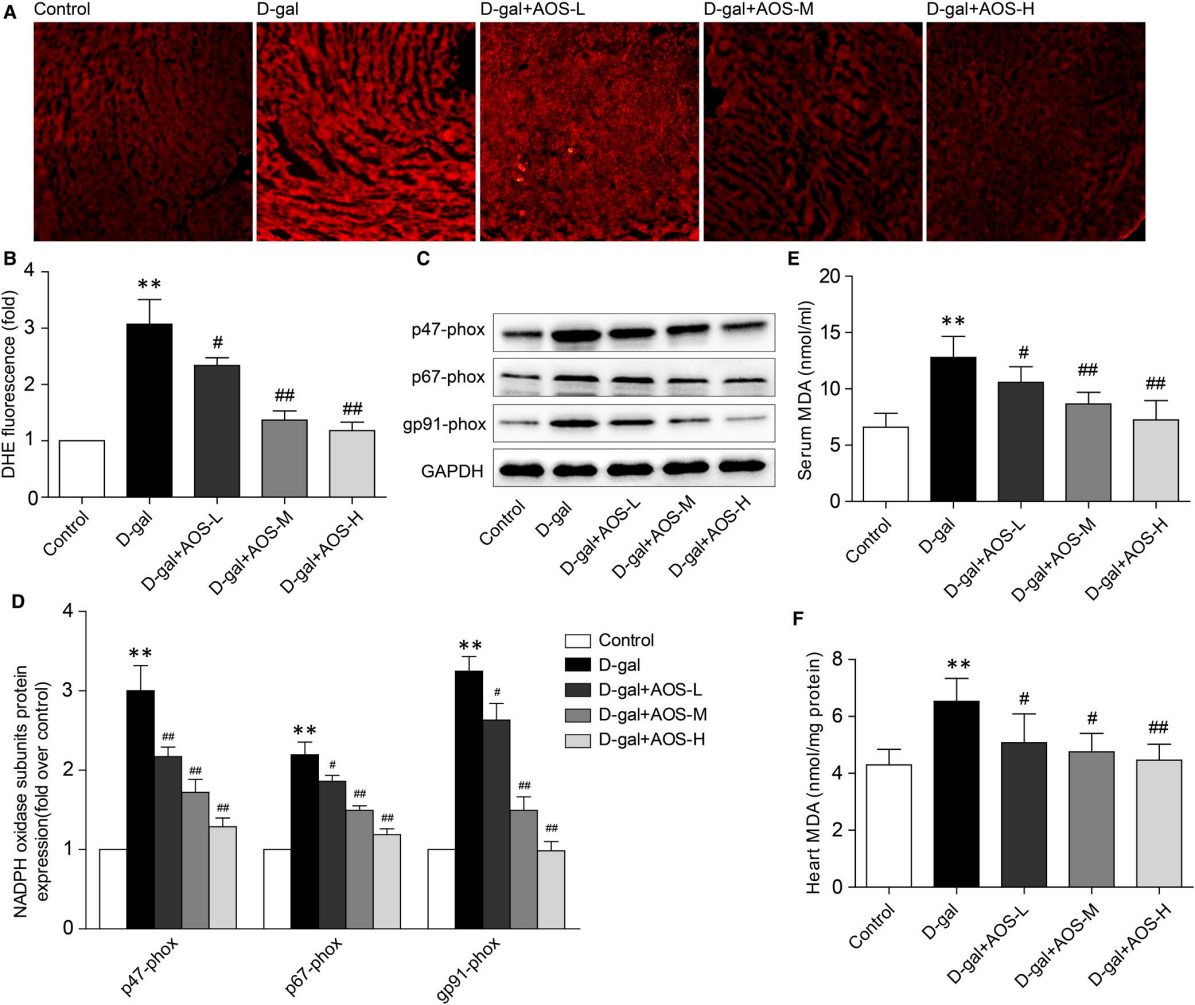
Alginate oligosaccharide decreased the ROS production and oxidative stress status in a dose‐dependent manner. (A) ROS generation was determined by dihydroethidium (DHE) fluorescence staining. (B) The quantitative data of (A). (C) The expression of NADPH oxidase subunits in heart tissues was determined by Western blot. (D) The corresponding quantitative data of (C). (E) Serum malondialdehyde (MDA) levels were evaluated. (F) Heart homogenates MDA levels were evaluated. **P < .01 vs Control mice, #*P* < .05 vs D‐gal mice, ##*P* < .01 vs D‐gal mice

We determined ROS production in the hearts using DHE fluorescence staining. As shown in Figure 6A‐B, we found that left ventricular cardiomyocyte DHE fluorescence intensity was significantly enhanced in D‐gal‐induced ageing mice, and AOS significantly decreased the left ventricular cardiomyocyte DHE fluorescence intensity.

To investigate the effect of AOS on oxidative stress status in the hearts of D‐gal‐induced ageing mice, we used Western blot to determine the protein expression of NADPH oxidase in heart tissues. The NADPH oxidase subunit p47‐phox, p67‐phox and gp91‐phox expression was significantly increased in D‐gal‐induced ageing mice. However, AOS decreased the expression of those NADPH oxidase subunits in a dose‐dependent manner (Figure 6C‐D).

Since MDA is considered a presumptive biomarker for lipid peroxidation in live organisms, furthermore, we detected MDA concentration to evaluate oxidative stress status in vivo. As shown in Figure 6E‐F, increased oxidative stress status in D‐gal‐induced ageing mice was confirmed by higher concentration of MDA in serum and heart homogenates. Administration of AOS decreased MDA levels in a dose‐dependent manner when compared with D‐gal group. Collectively, these data indicated that AOS significantly decreased the ROS production and oxidative stress status in D‐gal‐induced ageing mice.

## 4 DISCUSSION

Ageing is a chronic and multi‐organ‐related systemic changes of organism. The increasing age‐related cardiovascular diseases and the consequent financial burden have become a predominant worldwide challenge.^30^, ^31^ Therefore, the molecular mechanisms of cardiac ageing needs to be deeply studied, and novel anti‐ageing interventions will be of great significance in improving healthspan as well as delay cardiac insufficiency caused by ageing.

Alginate is a linear copolymer of polysaccharides extracted from brown sea algae. Due to its high molecular weight and viscosity, it is difficult for alginate to cross cell membranes and biological barriers, which limits its utilization. AOS, derived from the hydrolysis of alginate, has attracted increasing attention because of its lower molecular weight and viscosity. AOS is a water‐soluble, non‐toxic, non‐immunogenic and biodegradable compound and has much better biological activities than alginate.^7^ Evidence suggests that AOS, as an antioxidant oligosaccharide, has the abilities to protect neurodegenerative diseases and acute doxorubicin cardiotoxicity through inhibiting endoplasmic reticulum stress and oxidative stress.^5^, ^7^ Recent studies have demonstrated that marine‐derived AOS shows promising biological activity in reducing hyperlipidaemia, hyperglycaemia and hypertension and suppressing obesity.^27^ However, the ability of AOS to protect against ageing has not been demonstrated.

Previous studies indicate that ageing heart exhibit unique histological and functional features, including progressive cardiac remodelling and deteriorating cardiac reserve.^25^, ^32^ Intrinsic cardiac ageing may eventually result in increased vulnerability to various stressors and favouring the development of cardiovascular diseases.^33^ D‐gal‐induced premature ageing models display similar phenotypes of cardiac alterations compared with natural ageing in rodents.^34^ In this study, we demonstrated that D‐gal‐induced ageing mice exhibited abnormal myocardial architecture and increased collagen accumulation in myocardium interstitial and perivascular regions, and AOS administration inhibited the cardiac remodelling induced by D‐gal in C57BL/6J mice. Meanwhile, we found that AOS prevented cardiac dysfunction in D‐gal‐induced ageing mice, including partially preserved EF% and FS%, and decreased the expression of ANP and BNP. Echocardiography was also used to analyse the cardiac structure changes of mice. D‐galactose mice had a tendency to increase LVEDD and LVPWd, but there was no statistically significant difference compared with the control mice. However, LVESD was significantly increased in D‐gal‐induced ageing mice, and AOS decreased LVESD in a dose‐dependent manner. The reason for this phenomenon may be that the D‐galactose mimic accelerated ageing model has a relatively short modelling time, which mainly affects mitochondrial function as shown below and leads to cardiac dysfunction. Major heart structure changes were occurring but did not result in statistically significant differences. In addition, our data also showed that AOS administration significantly decreased the ageing markers p53 and p21 expression in a dose‐dependent manner. To further explore the possible mechanisms contributing to the anti‐ageing protective ability of AOS, the age‐related mitochondrial compromise was analysed.

The heart contains numerous mitochondria based on its extraordinary demand for ATP. Mitochondria generate ATP through oxidative phosphorylation of fatty acids and carbohydrate.^35^ In addition, free radicals are also constantly generated during this process. It is worth noting that mitochondrial homeostasis is vital for maintaining the function and viability of cardiomyocytes.^36^ Indeed, independent studies in the ageing heart have illustrated that cardiomyocyte mitochondria become impaired during ageing.^37^, ^38^ The ultrastructural morphometric analysis of aged rodents myocardium shows the presence of enlarged and swelling mitochondria, with the matrix derangement and loss of cristae.^39^ In this study, compared to the control mice, some cardiomyocyte mitochondria of D‐gal mice were enlarged, swelling and partial loss of cristae. AOS treatment alleviated the destruction of the cardiac mitochondrial ultrastructure in D‐gal‐induced ageing mice. In parallel, D‐gal induced a significant decrease in the level of cardiac MMP, and AOS administration protected against D‐gal‐mediated decline of MMP. These results indicated that AOS prevented the cardiac mitochondria from being destroyed in D‐gal‐induced ageing mice.

It has been noted that multiple compromised mitochondrial processes are implicated in the pathogenesis of aged heart, including the alteration of mitochondrial biogenesis and removal. Meanwhile, mitochondrial dysfunction and impaired removal of destroyed mitochondria, in turn, increase the vulnerability to stress injury of the aged heart.^40^ Mitochondrial biogenesis is mainly regulated by a set of nuclear encoded coactivators and transcription factors. Peroxisome proliferator–activated receptor γ coactivator 1α (PGC‐1α), which is critical for maintaining energy homeostasis, has been shown to play a key role in regulating mitochondrial biogenesis and function.^41^ Decreased PGC‐1α expression and activity have been linked to cardiac ageing and the development of heart failure.^42^ Previous study demonstrates that age‐related p53 activation directly results in mitochondrial compromise through repressing several master regulators of mitochondrial biogenesis, including PGC‐1α.^43^ Accumulating lines of evidence also suggest that the activation of PGC‐1α by pharmacological or genetic intervention prevents the ageing‐related changes in heart.^44^ Sirtuins (SIRTs) are recognized as non‐dispensable regulators of ageing process. SIRT3, localized in the mitochondrial matrix, plays an important role in maintaining mitochondrial bioenergetics, modulating mitochondrial metabolism and regulating lifespan. Sirt3 deficiency has been shown to lead to cardiac abnormalities and impair mitochondrial bioenergetics in mice. ^45^ In this study, we showed that a significant decrease in PGC‐1α and SIRT3 protein expression was observed in heart tissues of D‐gal‐induced ageing mice, and AOS significantly increased the PGC‐1α and SIRT3 protein expression in a dose‐dependent manner. Moreover, mtDNA probe PCR result showed that mtDNA copy number was significant decrease in D‐gal‐induced ageing mice, and AOS significantly increased the mtDNA copy number in a dose‐dependent manner. Taken together, these data indicated that AOS up‐regulated the mitochondrial biogenesis in D‐gal‐induced ageing mice.

As noted above, the efficient removal of impaired mitochondria is vital for the maintenance of cardiomyocytes homeostasis. The well‐known mechanism whereby mitochondria are turned over is through autophagy. It is proposed that autophagy plays a critical role in regulating cardiac homeostasis in basal state as well as in response to stress.^46^, ^47^ However, autophagy is generally down‐regulated in the ageing heart. Lower level of autophagy during the ageing process reduces the degradation of destroyed organelles and toxic proteins and induces the accumulation of dysfunctional and abnormal mitochondria, which eventually result in global cardiac dysfunction. Stimulation of autophagy has been demonstrated to improve cardiac function in rodent models by removing dysfunctional mitochondria, thereby optimizing the overall cellular environment and eventually alleviating ageing‐associated pathology in the heart.^48^ In this study, we demonstrated that the autophagy level significantly reduced in heart tissues of D‐gal‐induced ageing mice, and AOS significantly enhanced the autophagy level in a dose‐dependent manner. These changes revealed that AOS enhanced the efficient removal of age‐related impaired mitochondria through up‐regulating autophagy.

As previously discussed, the bulk of ROS production, generated from mitochondrial electron transport chain (ETC) complexes, occurs as a by‐product of mitochondrial oxidative phosphorylation.^44^ Experimental evidence indicates that the damaged mitochondria over the course of ageing generate increased amounts of ROS, enhance free radical–inflicted damage and eventually lead to a progressive forward‐feedback spiral of mitochondrial decay.^42^ This in turn induces more ROS through damage to the ETC, inducing further ROS production and ultimately leading to bioenergetics failure. In addition to the ETC, nicotinamide adenine dinucleotide phosphate (NADPH) oxidase is a major extramitochondrial sources of ROS, which might be responsible for oxidative stress during the ageing process and aggravate the oxidative damage to mitochondria. In addition to these mechanisms, recent study has also shown that lower mitochondrial ROS generation contributes to produce a low level of mtDNA steady‐state damage. ^49^ A higher mitochondrial base excision repair (BER) and a low mitochondrial damage of long‐live mammals in general contributes to their superior longevity. A recent report shows that NADPH oxidase inhibitor represses mitochondrial dysfunction in the ventral cochlear nucleus of D‐gal‐induced ageing rats.^50^ MDA is considered a presumptive biomarker for lipid peroxidation in live organisms, and MDA concentration can be used to evaluate oxidative stress status in vivo.^51^ In this study, we found that the administration of AOS attenuated ROS production, down‐regulated the expression of NADPH oxidase and decreased the level of MDA in D‐gal‐induced ageing mice. Our current results suggested that AOS efficiently decreased the oxidative stress status in D‐gal‐induced ageing mice, which might be another important protective factor for AOS to alleviate mitochondrial damage.

In the present study, we first clarified that AOS alleviates D‐gal‐induced cardiac ageing through improving mitochondrial biogenesis, maintaining the mitochondrial integrity and enhancing the efficient removal of impaired mitochondria in C57BL/6J mice. In addition, AOS also decreases the ROS production and oxidative stress status, which, in turn, further inhibit cardiac mitochondria from being destroyed. AOS may be an effective therapeutic agent to alleviate cardiac ageing. In this study, we only used male mice and did not examine sex differences on the effects of AOS. It is very interesting that dendritic cell–specific intercellular adhesion molecule‐3‐grabbing non‐integrin (DC‐SIGN) ligand 1 (DSCL1, an anti‐inflammatory agent) treatment reduces macrophage polarization and diastolic dysfunction in the ageing female but not male mouse heart.^52^ This paper suggests that it would be of great significance to compare sex differences in future studies.

In conclusion, our findings suggested that AOS alleviated D‐gal‐induced cardiac ageing via regulating myocardial mitochondria function and integrity in mice. AOS exhibits a wide range of promising biological activities and may be an effective therapeutic agent to alleviate cardiac ageing.

## CONFLICT OF INTEREST

The authors confirm that there are no conflicts of interest.

## AUTHOR CONTRIBUTION

**Wenjing Feng:** Conceptualization (lead); Data curation (lead); Formal analysis (lead); Funding acquisition (lead); Project administration (lead); Software (lead); Supervision (lead); Writing‐original draft (lead); Writing‐review & editing (equal). **Jianya Liu:** Data curation (equal); Formal analysis (equal); Software (equal). **Shan Wang:** Data curation (supporting); Methodology (supporting); Visualization (supporting). **Yi Hu:** Conceptualization (supporting); Formal analysis (supporting); Investigation (supporting); Validation (supporting). **Hui Pan:** Data curation (supporting); Writing‐original draft (supporting). **Ting Hu:** Data curation (supporting); Software (supporting); Validation (supporting). **Huashi Guan:** Supervision (supporting); Validation (equal); Writing‐review & editing (supporting). **Dongfeng Zhang:** Conceptualization (supporting); Methodology (supporting); Supervision (supporting); Validation (supporting); Writing‐review & editing (supporting). **Yongjun Mao:** Conceptualization (lead); Data curation (lead); Funding acquisition (lead); Project administration (lead); Supervision (lead); Writing‐review & editing (lead).
